# Single-cell transcriptome analysis reveals T-cell exhaustion in denosumab-treated giant cell tumor of bone

**DOI:** 10.3389/fimmu.2022.934078

**Published:** 2022-09-12

**Authors:** Meiling Yang, Fen Wang, Guohao Lu, Mingzhe Cheng, Wei Zhao, Changye Zou

**Affiliations:** ^1^ Guangdong Cardiovascular Institute, Guangdong Provincial People’s Hospital, Guangdong Academy of Medical Sciences, Guangzhou, China; ^2^ Pathologica Department, The First Affiliated Hospital of Sun Yat-Sen University, Guangzhou, China; ^3^ Key Laboratory of Stem Cells and Tissue Engineering (Sun Yat-Sen University), Ministry of Education, Guangzhou, China; ^4^ Musculoskeletal Oncology Department, the First Affiliated Hospital of Sun Yat-Sen University, Guangzhou, China

**Keywords:** Denosumab, giant cell tumor of bone, T-cell exhaustion, periostin, single-cell RNA-seq, RANKL

## Abstract

Denosumab (DMAB), a human monoclonal antibody against the receptor activator of the nuclear factor-kappa B ligand, is used for the treatment for unresectable giant cell tumor of bone (GCTB). However, little is known about the molecular and functional characteristics of GCTB-infiltrating lymphocytes after DMAB treatment. Here, we performed single-cell RNA sequencing and immunostaining assays to delineate the immune landscape of GCTB in the presence and absence of DMAB. We found that exhausted CD8^+^ T cells were preferentially enriched in DMAB-treated GCTB. A distinct M2-skewed type of tumor-associated macrophages (TAMs) comprises the majority of GCTB TAMs. We identified cytokines, including interleukin-10, and inhibitory receptors of M2 TAMs as important mediators of CD8^+^ T cell exhaustion. We further revealed that DMAB treatment notably increased the expression levels of periostin (POSTN) in GCTB cells. Furthermore, POSTN expression was transcriptionally regulated by c-FOS signaling and correlated with GCTB recurrence in patients after DMAB treatment. Collectively, our findings reveal that CD8^+^ T-cells undergo unappreciated exhaustion during DMAB therapy and that GCTB cell-derived POSTN educates TAMs and establishes a microenvironmental niche that facilitates GCTB recurrence.

## Introduction

Giant cell tumor of bone (GCTB) is a locally aggressive bone tumor. It is mainly composed of two cell types: active osteoclast-like giant cells expressing the receptor activator of nuclear factor-kappa B (RANK), and neoplastic mononuclear stromal cells expressing the RANK ligand (RANKL) (1–3). The standard treatment for GCTB is the surgical resection of the tumor, including en bloc resection and extensive curettage. Curettage combined with local adjuvants should be the first choice for preserving a functional joint, although it shows a high recurrence rate of GCTB (4). Denosumab (DMAB), a human monoclonal antibody against RANKL, has been approved by the US Food and Drug Administration and the European Medicines Agency, and specifically inhibits RANKL-mediated formation and activation of osteoclast-like giant cells (4–6). Numerous clinical trials have shown that DMAB is correlated with a beneficial tumor response, but there are many controversies regarding its safety (5, 7–11). Moreover, the exact molecular basis and factors affecting the efficacy of DMAB remain poorly understood.

In addition to promoting osteoclast formation and activation, the RANKL–RANK signaling pathway plays important roles in lymph node development (12), lymphocyte differentiation, T-cell activation, dendritic cell survival, and immune tolerance induction (13, 14). RANKL-deficient mice do not exhibit lymph node metastasis (15). During initial T-cell receptor activation, T cells may provide RANKL directly to dendritic cells (DCs) to promote long-term interactions. In the absence of RANKL–RANK engagement, DCs may be at an increased risk of apoptosis, leading to reduced T cell activation (16–19). Thus, inhibition of RANKL could increase the immune escape as a result of T cell inactivation. Moreover, RANK expression in tumor-associated myeloid immune cells, such as DCs, TAMs, and myeloid-derived suppressor cells, is observed in diverse mouse tumor models and human tumors. The blocking of the RANKL–RANK interaction could either promote or suppress the antitumor immunity, depending on the specific phase and activated pathways (20). The potential effects of DMAB on the functions of myeloid immune cells present in the tumor microenvironment (TME) of GCTB remain unclear.

Single-cell RNA sequencing (scRNA-seq) has recently been used to characterize subsets of immune cells in tumors and their corresponding transcriptome changes upon treatment (21–24). Previous scRNA-seq data related to GCTB revealed the heterogeneity of osteoclasts and immune cells in GCTB (25). In the present study, we used scRNA-seq and immunostaining assays to detect the dynamic changes in the immune cells in GCTB and to unveil CD8^+^ T-cell exhaustion associated with DMAB therapy.

## Materials and methods

### Human GCTB samples

This study was approved by the Medical Ethics Committee of the First Affiliated Hospital of Sun Yat-Sen University. All patients provided informed consent preoperatively. For scRNA-seq, two patients with or without DMAB treatment were pathologically diagnosed with GCTB at the First Affiliated Hospital of Sun Yat-Sen University. Formalin-fixed paraffin embedded (FFPE) archival tissue blocks used for Immunohistochemistry (IHC) and immunofluorescence (IF) staining assays were collected from GCTB tumors with or without DMAB treatment.

### Single-cell isolation and scRNA-seq

Fresh tumor tissues were surgically removed from GCTB patients and minced into 2-4 mm pieces. The pieces were transferred to the tube with digestive enzyme from Tumor Dissociation Kit (Cat# 130-095-929, Miltenyi Biotec) and incubated at 37 °C for 30 min on a shaker. After digestion, 2% FBS was added to neutralize enzyme lysate, and the tissues were filtered through a 70 μm filter. Subsequently, the samples were centrifuged at 350 × g for 5 min and the supernatants were discarded. To remove red blood cells, the cell pellets were suspended in red blood cell lysis buffer (Beyotime) for 30 s. The solution was then centrifuged at 350 × g for 5 min and resuspended in Dulbecco’s phosphate-buffered saline (DPBS; Thermo Fisher). The samples were stained with trypan blue (Solarbio) and the cellular viability was evaluated. Finally, single cells were encapsulated into emulsion droplets using the Chromium Controller (10× Genomics). The scRNA-seq libraries were constructed following the manufacturer’s instructions (10× Genomics) and then used for sequencing.

### Pre-processing of scRNA-seq data

Raw data were processed using Cell Ranger (v3.0.2) to align reads, generate feature-barcode matrices, and perform gene expression analysis. We used the mkfastq pipelines to make fastq files and used the cell count pipelines for alignment (with reference genome Hg19), filtering, barcode counting, and UMI counting.

The raw output data were processed with the Seurat package (version 4.0.4; http://satijalab.org/seurat/) in R software. We filtered out the cells with less than 200 genes and the percent of mitochondrial genes over 25% of total expressed genes. Genes detected in fewer than 10 cells were also excluded. Feature counts for each cell were divided by the total counts for that cell and multiplied by the scale factor (10,000), and then natural-log transformed (default Seurat approach). To adjust for technical variation and batch effects between samples, we used the standard anchor-based workflow for dataset integration in Seurat (26). The merged dataset included 13,857 cells and 20,438 detected genes across the two samples. Integration-transformed expression values were used only for dimension reduction and clustering. The original lognormalized expression values were used for all differential expression and gene set level analyses.

### Unsupervised clustering and identification of cell types

We used the Seurat package to perform unsupervised clustering. We performed principal component analysis (PCA) on the integration-transformed expression matrix using highly variable genes identified by ‘‘FindVariableFeatures’’ function. Following the results of PCA, the appropriate principal components (PCs) were selected for clustering with the specific resolution parameters. The identified clusters were visualized on the 2D map produced with the t-SNE method. The cell groups were annotated based on the well-known cellular markers from the literature (27, 28).

For the clustering of all cells, the top 15 PCs were selected with a resolution parameter equal to 0.8. For the clustering of myeloid cells, the top 15 PCs were selected with a resolution parameter equal to 0.6. For the clustering of TILs, we used the top 15 PCs with a resolution parameter equal to 2, but a group of cells expressed both macrophage and T cell marker genes. We eliminated this group of cells because it was most likely doublets and then reclustering TILs with the top 15 PCs and a resolution parameter equal to 1.2.

### Identification of tumor cells

Tumor cells were identified using cluster-level marker genes expression (BGLAP, RUNX2, TNFSF11, IBSP) and inferred CNV profiles. We performed InferCNV (inferCNV of the Trinity CTAT Project. https://github.com/broadinstitute/inferCNV) within each sample, using TILs as reference group and tumor cells as observation group. We identified large-scale chromosomal copy number variants, either gains or losses, in tumor cells, in addition to the expression of RUNX2. The raw single-cell gene expression data was extracted from the Seurat object according to the software recommendation. The inferCNV analysis was performed with default parameters including a value of 0.1 for “cutoff”.

### IHC and IF staining assays

IHC and IF staining of FFPE GCTB specimens were performed according to the previous published paper (29). Briefly, all sections were deparaffinized, rehydrated and antigen repaired. For IHC staining of RANKL, RUNX2 and POSTN, endogenous peroxidase was blocked using 3% hydrogen peroxide for 30 min. Samples were blocked with goat serum at room temperature (RT) for 1 h and incubated separately with RANKL antibody (Cell Signaling Technology, 1:200), RUNX2 antibody (ab76956, Abcam, 1:200), RANK antibody (Cell Signaling Technology) and POSTN antibody (ab14041, Abcam, 1:800) overnight at 4°C, followed by horseradish peroxidase (HRP)-linked secondary antibodies and DAB staining (#8059, Cell Signaling Technology). Counterstaining was done with hematoxylin.

The rate of RANKL-, RANK-, or RUNX2-positive cells was counted, based on all cells of the whole tissue slide. The scoring system of POSTN staining was blind observed at 5 fields of the sections under a 20-fold microscope, and scored the immunohistochemical staining intensity and positive areas. Staining intensity score: 0 points for no staining, 1 point for light yellow, 2 points for tawny and 3 points for brown; Positive area percentage score: 0 points for no positive area, 1 point for < 30%, 2 points for 30%~60%, and 3 points for more than 60%. The product of the score values was used as the score of immunostaining score, with a total of 7 score level (0, 1, 2, 3, 4, 6, and 9). The patients were divided into high expression group and low expression group according to the median patient score.

For IF staining of CD8 and LAG3, samples were blocked with 5% donkey serum at RT for 1 h and incubated with CD8 antibody (ab199016, Abcam, 1:100) and LAG3 antibody (ab180187, Abcam, 1:5000) overnight at 4°C. After washing, samples were incubated for 1 h at RT with fluorescently labeled secondary antibodies including donkey anti-mouse Alexa Fluor 488 (ab150105, Abcam, 1:500) and donkey anti-rabbit Alexa Fluor 555 (ab150074, Abcam, 1:500). Nuclei were counterstained with Hoechst33342 (H3570, Thermo Fisher Scientific, 1:500). To remove unwanted fluorescence in tissue sections due to aldehyde fixation, red-blood cells, and structural elements, we used Vector TrueVIEW Autofluorescence Quenching Kit (SP-8400, VECTOR). To ensure representativeness, the whole tissue slide was observed, and 5 fields with high infiltration of CD8^+^ T cells were selected for photographing with laser confocal scanning microscope (LSM780) and the rate of CD8^+^LAG3^+^ T cells in CD8^+^ T cells was calculated.

### Cell-cell interaction analysis

To analyze cell-cell interactions between different cell types, we used CellChat (version 1.1.2; https://github.com/sqjin/CellChat/) (30) to identify significant ligand-receptor pairs within no DMAB and DMAB samples. For both no DMAB and DMAB samples, the cell type specific ligand-receptor interactions were identified based on the specific expression of a receptor by one cell type and a ligand by another cell type. We used the “netVisual_bubble” function to show the cell communication mediated by exhaustion-related ligand-receptor pairs, and the “netVisual_chord_gene” function to visualize cell-cell communication for the enhanced signaling pathways to macrophages in GCTB after DMAB treatment.

### Definition of cell scores and signature

To evaluate the potential functions of a cell group of interest from No DMAB and DMAB samples, we calculated the scores of functional feature sets for the cell group, using the “AddModuleScore” function in Seurat at single cell level. The average expression levels of the functional feature sets were subtracted by the aggregated expression of control feature sets. All analysed genes were binned based on averaged expression, and the control features were randomly selected from each bin.

The functional gene sets including M1/M2 polarization and anti-inflammatory for macrophages, co-stimulatory and exhausted for CD8^+^ T cells. The involved gene sets were listed in the supplementary material (**Supplementary Table S1**) (27).

### Trajectory analysis of single cells

The single-cell pseudotime trajectories were generated with the Monocle package (v2.20.0) in R (31–33). The gene-cell matrix in the raw counts derived from the Seurat RNA assays were used as the inputs. The “newCellDataSet” function was applied to create an object for subsequent analysis. We Filtered out low-quality genes by using “detectGenes” function with the parameters “min_expr = 0.1”. We reduced data dimensionality by using the “reduceDimension” function with the parameter reduction_method = “DDRTree”. The cells were ordered and visualized with the “plot_cell_trajectory” function. Genes of interest that changed along with the pseudotime were visualized with the “plot_pseudotime_heatmap” and the genes were clustered into subgroups according to the gene expression patterns. To identify the genes that separate cells into branches, the branch expression analysis modeling (BEAM) analysis were performed. Genes of interest, resulting from the BEAM analysis, were visualized with the “plot_genes_branched_heatmap” function and “plot_genes_branched_pseudotime” function. In the single-cell pseudotime trajectories analysis of CD8^+^T cells, we excluded the proliferative subgroup CD8 Ki67, because its high proliferative status would affect the trajectory analysis.

### DEGs identification

DEGs of tumor cells between No DMAB and DMAB samples were identified using “FindMarkers” functions in Seurat with the Wilcoxon Rank-Sum test (logfc.threshold = 0.25).

### ChIP-qPCR assay

GCTB tissues from three patients were cross-linked with 1% (v/v) formaldehyde at room temperature for 10 min and then processed using the Magna ChIP G-Chromatin Immunoprecipitation Kit (Sigma-Aldrich, 17-611) according to manufacturer’s instructions. The subsequent qPCR was conducted using the 2 × ChamQ Universal SYBR qPCR Master Mix (Vazyme, China). The primer sequences for POSTN were as follows: forward, TGAGACTTAAACATGCAGTGAGT; Reverse, ACATTGAGCTACTTTTCCTTTTCAT.

### Statistics

Comparisons of gene expression or functional signature between two groups of cells were performed using unpaired two-tailed student’s t test. Statistical analyses and presentation were performed using R. Comparisons of the fractions of RANKL^+^ or RUNX2^+^ cells in paired No DMAB and DMAB were performed using wilcoxon matched-pairs signed rank tests. Comparisons of the infiltration ratio of CD8^+^LAG3^+^ T cells in unpaired No DMAB and DMAB were performed using unpaired two-tailed t test. Statistical analyses and presentation were performed using Graphpad prism8. Other statistical tests used in figures were shown in figure legends.

## Results

### scRNA-seq profiling of the TME in GCTB with or without DMAB treatment

**Figure 1:**
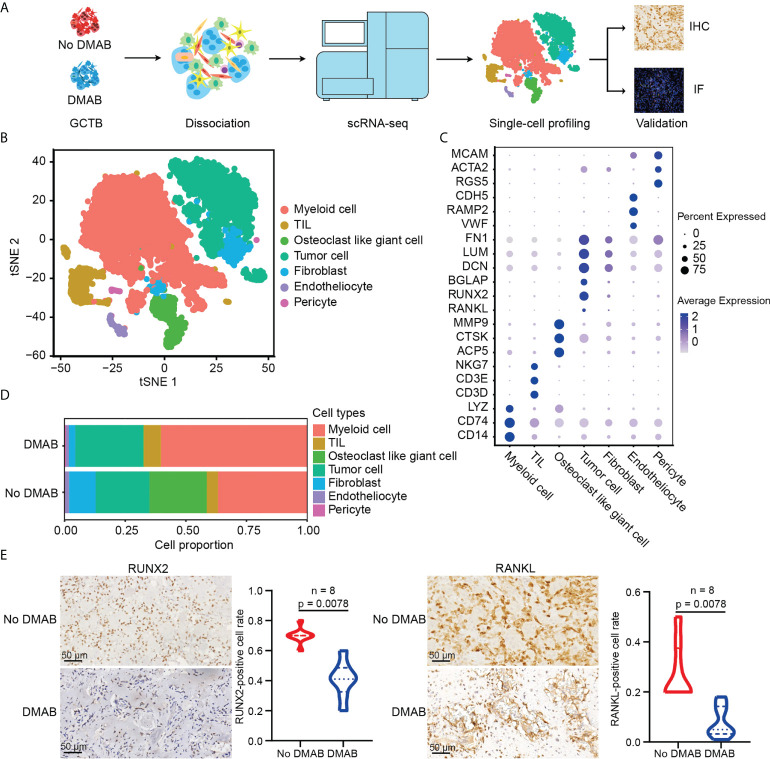
Single-cell RNA sequencing (scRNA-seq) profiling of the untreated and DMAB-treated giant cell tumor of bone (GCTB) tumor microenvironments (TMEs). **(A)** Schematic representation of the experimental design. Single-cell suspensions were collected from GCTB tumors of two patients followed by scRNA-seq on 10× Genomics platform. A total of 13,857 qualified single cells were recovered. **(B)** T-distributed stochastic neighbor embedding (t-SNE) plot showing the annotation and color codes for cell types in the GCTB ecosystem. **(C)** Dot plot showing the expression levels of marker genes in the indicated cell types. The size of the dot represents the proportion of cells expressing the particular marker within the group and the spectrum of color indicates the mean expression levels of the markers. Blue color dots indicate the highly expressed genes, while gray color dots indicate the low expressed genes. **(D)** Histogram showing the proportion of all cell types in untreated and denosumab (DMAB)-treated samples. **(E)** Representative images of immunohistochemistry (IHC) staining in formalin-fixed paraffin-embedded (FFPE) tissues, indicating RUNX family transcription factor 2 (RUNX2) ^+^ cells and RANKL^+^ cells in paired No DMAB and DMAB samples (n = 8). Scale bar, 50 μm. Violin plot presenting the fractions of RUNX2^+^ cells and RANKL^+^ cells in paired No DMAB and DMAB samples based on IHC staining results. Statistical analyses are paired Wilcoxon tests.

To reveal the TME in DMAB-treated GCTB, we collected surgical tumor specimens from untreated (No DMAB) and DMAB-treated (DMAB) GCTB patients for scRNA-seq. After the initial quality control assessment, single-cell transcriptomes were obtained from 13,857 cells. In addition, there were 8 paired GCTB samples in immunohistochemistry (IHC) assays for validation of RANKL and RUNX family transcription factor 2 (RUNX2) expression, 6 unpaired GCTB samples (3 treated vs. 3 untreated) in IF assays for validation of CD8 and lymphocyte activating 3 (LAG3) expression, and 32 GCTB samples (9 no DMAB vs. 23 DMAB; 14 no recurrence vs. 9 recurrence within 23 DMAB) in IHC assays for validation of POSTN expression (**Figure 1A**).

Seurat was used for cell classification and marker gene identification. Seven main clusters were identified and visualized using the t-distributed stochastic neighbor embedding (t-SNE) method (**Figure 1B**). They were as follows (1): myeloid cells highly expressing CD74, CD14, and lysozyme (2); TILs, including T and NK cells, specifically expressing CD3D, CD3E, and NKG7 (3); osteoclast-like giant cells with high expression of ACP5, CTSK, and MMP9 (4); tumor cells highly expressing BGLAP, RUNX2, RANKL, and IBSP (5); fibroblasts specifically expressing FN1, LUM, and DCN (6); endotheliocytes specifically expressing VWF, RAMP2, and CDH5; and (7) pericytes highly expressing RGS5, ACTA2, and MCAM (**Figure 1C** **;** **Supplementary Figure S1A**). To ensure the correct definition of tumor cells, we applied the inferCNV algorithm to analyze the copy number variations (CNVs) of tumor cells using TILs as control cells (data not shown), and confirmed that the defined tumor cells had obvious CNVs (**Supplementary Figure S1B**).

We noticed that almost all types of cell populations were present in both DMAB-treated and untreated samples, except for osteoclast-like giant cells, which were significantly missing in the DMAB sample (**Figure 1D**). In addition, we found that the expression of osteoblast-related genes RUNX2 and RANKL was reduced in tumor cells after DMAB treatment (**Figure 1E** **;** **Supplementary Figure S1C**). The expression of RANK, receptor of RANKL, was not significantly changed after DMAB treatment (**Supplementary Figures S1D, E**).

### DMAB treatment is associated with the exhaustion of CD8^+^ LAG3^+^ T cells in GCTB

**Figure 2:**
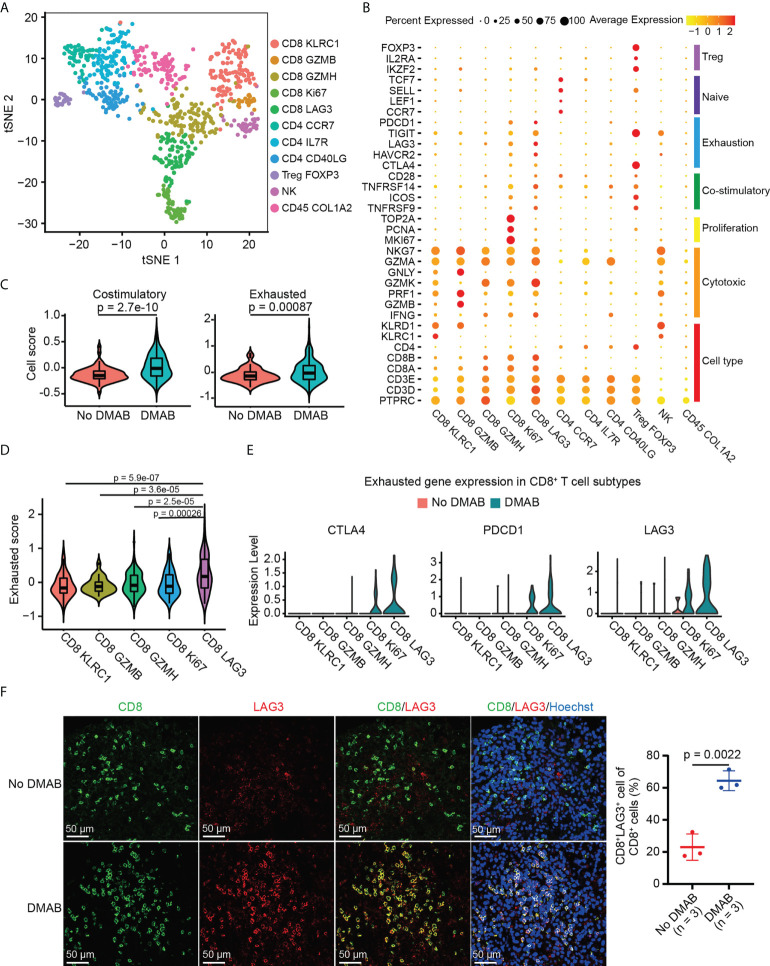
DMAB treatment promotes the exhaustion of CD8^+^ lymphocyte activating 3 (LAG3) ^+^ T cells in GCTB. **(A)** t-SNE plot showing the subtypes of tumor-infiltrating lymphocytes (TILs) derived from untreated and DMAB-treated patients with GCTB. **(B)** Dot plot showing the expression levels of selected gene sets in each subtype of TILs, including Treg, naive, exhaustion, costimulatory, proliferation, and cytotoxic cell types. **(C)** Violin plot showing the costimulatory and exhausted scores of CD8^+^ T cells from the No DMAB (red) and DMAB (blue) samples. The p values are calculated by Student’s *t* test. **(D)** Violin plot indicating the exhausted scores in CD8^+^ T cell subtypes. The p values are calculated by Student’s *t* test. **(E)** Violin plot showing the expression levels of selected exhausted genes in CD8^+^ T cell subtypes. Red, No DMAB; blue, DMAB. **(F)** Immunofluorescence **(IF)** staining of CD8 and LAG3 antibodies, showing the infiltration of CD8^+^LAG3^+^ T cells in unpaired patients with GCTB with (n = 3) or without (n = 3) DMAB. Scale bars = 50 μm. Based on the IF staining results, the infiltration ratio of CD8^+^LAG3^+^ T cells was statistically analyzed using an unpaired *t* test.

The re-clustering of TILs revealed 11 populations, including five subtypes of CD8^+^ T cells (CD8 KLRC1, CD8 GZMB, CD8 GZMH, CD8 Ki67, and CD8 LAG3), four clusters of CD4^+^ T cells (CD4 CCR7, CD4 IL7R, CD4 CD40LG, and Treg FOXP3), one NK subtype, and one unknown cluster with high COL1A2 expression (CD45 COL1A2) (**Figure 2A**). CD8^+^ T cells were identified by the high expression levels of CD3D and CD8A. They also highly expressed the genes associated with cytotoxicity (GZMA, GZMK, and NKG7). CD8 Ki67 cells displayed high expression of proliferative genes (MKI67, PCNA, and TOP2A) and moderate expression of exhaustion-related markers (LAG3, TIGHT, and PDCD1), suggesting that these cells represent an early exhausted state. CD8 LAG3 cells showed the highest expression levels of T cell exhaustion markers (LAG3, TIGHT, PDCD1, HAVCR2, and CTLA4), suggesting that these cells were exhausted (**Figures 2B** **;** **Supplementary Figure S2A**). In addition, we found that cytotoxic CD4^+^ T cells had increased infiltration and enhanced toxicity following DMAB treatment (**Supplementary Figures S2B, C**).

To further investigate the effect of DMAB on the functional characteristics of CD8^+^ T cells, we calculated the costimulatory and exhaustion scores of CD8^+^ T cells by analyzing the expression of related genes. Interestingly, we found that costimulatory and exhausted scores and related genes increased significantly in DMAB-treated samples compared to untreated GCTB (**Figure 2C** **;** **Supplementary Figures S2D, E**). Additionally, the number of exhausted CD8^+^ cells that expressed high exhaustion scores (**Figure 2D**) was higher in DMAB-treated samples than in untreated samples (**Figure 2E**). LAG3 is a recently recognized immune checkpoint, and its high expression correlates with T-cell exhaustion. We further verified the increased abundance of exhausted CD8^+^ T cells (CD8^+^LAG3^+^) in DMAB versus No DMAB using IF staining (**Figure 2F**). These results suggest that DMAB treatment correlates with CD8^+^ T cell exhaustion in GCTB.

### Dynamic cell transitions of CD8^+^ T cells in GCTB with or without DMAB treatment

**Figure 3:**
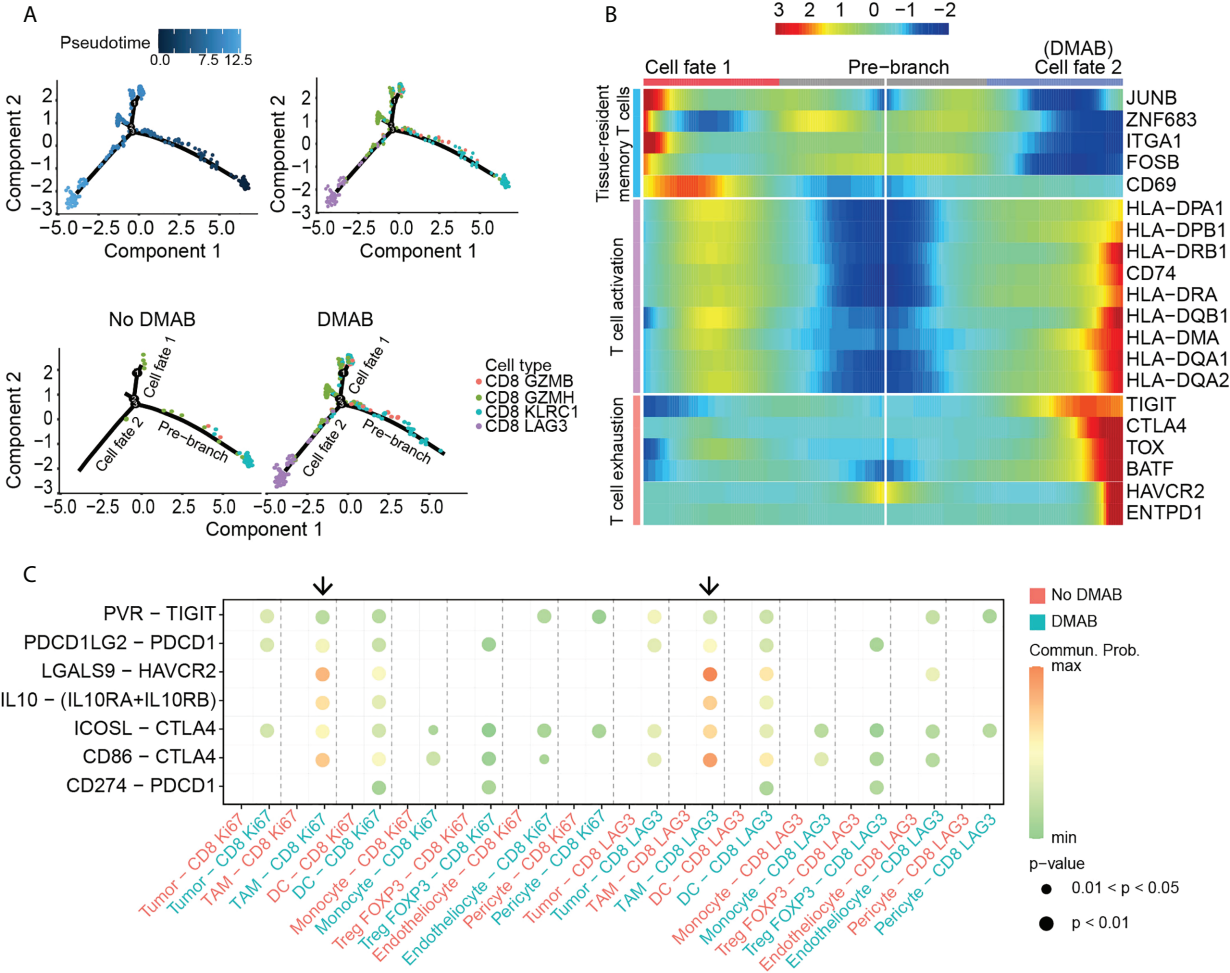
TAM induces the exhaustion of CD8^+^ T cells after DMAB treatment. **(A)** Pseudotime-ordered analysis of CD8^+^ T cells from No DMAB and DMAB samples. CD8^+^ T cell subtypes are labeled with colors. Each dot indicates a single cell. **(B)** Pseudotime heatmap showing the dynamic changes in gene expression along cell fate 1 and cell fate 2 according to the BEAM analysis. The selected genes are associated with tissue-resident memory T cells, T cell activation, and T cell exhaustion. **(C)** Bubble chart showing exhaustion-related signaling targeting exhausted subtypes (CD8 Ki67 and CD8 LAG3) based on selected ligand and receptor pairs, as calculated by CellChat. Dot color reflects communication probabilities and dot size represents computed p-values. Empty space means that the communication probability is zero. Black arrows indicate the communication between TAMs and exhausted subtypes in the DMAB sample (blue). The p values were computed using a one-sided permutation test.

Next, we explored the dynamic cell transitions and immune states in CD8^+^ T cells by inferring state trajectories using Monocle. This analysis showed that CD8 KLRC1 cells were at the beginning of the trajectory, whereas CD8 LAG3 cells were in a terminal state (**Figure 3A**, upper part). We identified three sets of differentially expressed genes along the CD8^+^ T cell trajectory. The first set, consisting of naive T cell markers (CCR6 and LEF1), decreased along the trajectory, while the second set, consisting of effector genes (IFNG) and cytotoxic genes (GZMB, GZMK, and GZMA), increased from the middle to the end of the trajectory. The last set, consisting of exhausted markers (LAG3, CTLA4, TIGHT, and PDCD1), increased towards the end of the trajectory (**Supplementary Figures S3A, B**). CD8^+^ T cells in the No DMAB sample only occupied branch one (cell fate 1), whereas CD8^+^ T cells in the DMAB sample covered the entire trajectory, including the two branches (cell fate 1 and cell fate 2) (**Figure 3A**, lower part). Based on BEAM, we found that tissue-resident memory T cell-related genes (CD69, ITGA1, and FOSB) were upregulated along cell fate 1, suggesting that this branch shifted towards memory T cells. Activation genes (HLA-DMA, HLA-DPA1, HLA-DQA1, and HLA-DRA) and exhausted genes (CTLA4, TOX, and ENTPD1) were both upregulated along cell fate 2 (**Figure 3B** **;** **Supplementary Figure S3C**), suggesting that CD8^+^ T cells in this branch were first activated and then subjected to exhaustion.

To determine the mechanism that induces CD8^+^ T cell exhaustion, we used CellChat for cell communication analysis. The analysis showed that DMAB treatment enhanced multiple exhaustion-related pathways, including PVR–TIGIT, PDCD1LG2-PDCD1, LGALS9-HAVCR2, IL10-IL10R and CD86-CTLA4, in various cell populations (**Figure 3C**). Intriguingly, TAMs targeted the exhausted subpopulation with the highest communication probability in the DMAB sample, suggesting that TAMs may contribute to the exhaustion of CD8^+^ T cells after DMAB treatment.

### Periostin enhances the M2-like phenotype of TAMs in DMAB treated GCTB

**Figure 4:**
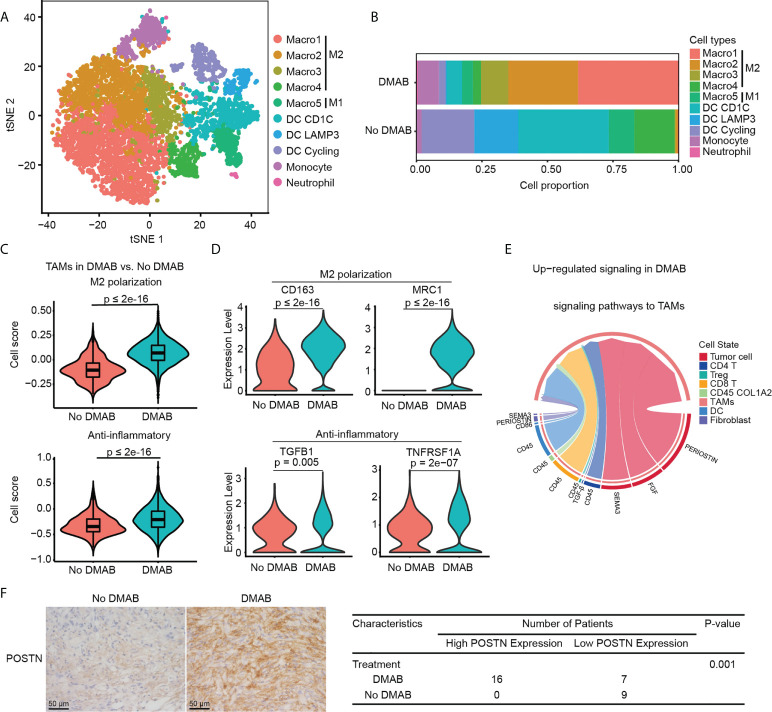
DMAB treatment promotes the M2 subtype of TAMs *via* periostin (POSTN). **(A)** t-SNE plot showing the subtypes of myeloid-derived cells derived from No DMAB and DMAB-treated GCTB patients. **(B)** Histogram showing the proportion of myeloid subgroups in No DMAB and DMAB-treated samples. **(C)** Violin plot showing the M2 polarization and anti-inflammatory scores of TAMs from the No DMAB (red) and DMAB-treated (blue) samples. The p values were calculated using a Student’s *t* test. **(D)** Violin plot showing the expression levels of selected M2 polarization and anti-inflammatory genes in TAMs from the No DMAB (red) and DMAB-treated (blue) samples. The p values were calculated using a Student’s *t* test. **(E)** Chord diagram showing the significantly upregulated signaling pathways in TAMs in GCTB after DMAB treatment. **(F)** Representative IHC images of POSTN in GCTB without DMAB (n =9) versus GCTB with DMAB (n = 23). Scale bar, 50 μm. Statistical table of 32 GCTB samples by POSTN staining intensity. Significance was determined by a fisher exact test.

Next, we performed unsupervised clustering of myeloid cells in GCTB. Ten clusters emerged within the myeloid lineage, including five clusters for TAMs (Macro1–Macro5), three for DCs (DC CD1C, DC LAMP3, and DC Cycling), one for monocytes, and one for neutrophils (**Figure 4A**). TAMs were identified by the high expression levels of CD68 and CD163. We could not clearly distinguish M1 and M2 TAMs using known marker genes, CD86 (M1) and CD163 (M2), as they were both expressed in these cells (**Supplementary Figure S4A**). However, by calculating M1 and M2 scores for each cell using related gene sets (**Supplementary Table S1**), we observed that clusters Macro1-Macro4 exhibited an M2-like phenotype, whereas cluster Macro5 exhibited an M1-like phenotype (**Supplementary Figure S4B**). DCs were identified by the expression of CD1C and CD1A. DC cycling showed high expression of proliferation-related genes (MKI67 and TOP2A) (**Supplementary Figure S4A**).

We found that the TAM subpopulations showed treatment bias. The proportion of TAMs was significantly higher in DMAB than in No DMAB samples, especially in M2-like TAMs (**Figure 4B**). To further investigate the effect of DMAB on the functional characteristics of TAMs, we calculated the M2 polarization and anti-inflammatory scores of TAMs by analyzing the expression of related genes. We found that the M2 polarization and anti-inflammatory scores increased significantly after DMAB treatment (**Figures 4C**). Consistently, the expression of M2 related genes (e.g., CD163 and MRC1) and anti-inflammatory related genes (e.g., TGFB and TNFRSF1A) was higher in DMAB-treated GCTB than in untreated GCTB (**Figure 4D**).

To determine the underlying mechanism by which GCTB facilitates the M2-like phenotype in TAMs, we used CellChat to analyze cell communication between TAMs and GCTB cells. We found that the POSTN pathway (POSTN-ITGAV/ITGB5) was enhanced from GCTB tumor cells to TAMs after DMAB treatment (**Figure 4E** **;** **Supplementary Figure S4C**). In addition, POSTN expression levels were upregulated in DMAB-treated GCTB compared to untreated control (**Figure 4F**). These analyses indicated that POSTN secreted by GCTB cells likely promoted the M2-like phenotype in TAMs.

### POSTN expression is associated with the relapse of GCTB after DMAB treatment

**Figure 5:**
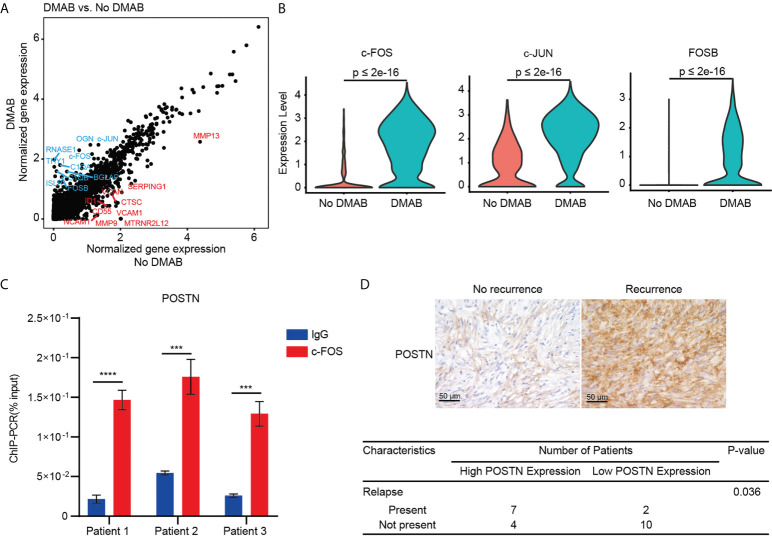
C-FOS-mediated POSTN expression is associated with the relapse of GCTB after DMAB treatment. **(A)** Scatter plot showing the DEGs of tumor cells from DMAB-treated sample versus No DMAB sample. The top 10 DEGs were labeled in blue (upregulated in DMAB-treated samples) or in red (upregulated in No DMAB samples). **(B)** Violin plot showing the expression levels of activator protein 1 (AP-1) transcription factor (c-FOS, c-JUN, and FOSB) in tumor cells from the No DMAB (red) and DMAB-treated (blue) samples. The p values were calculated using a Student’s t test. **(C)** ChIP-qPCR analysis of c-FOS enrichment at the POSTN promoter region in 3 GCTB tissues. Significance was determined by a Student’s t test. **(D)** Representative IHC images of POSTN in GCTB without recurrence (n = 14) versus GCTB with recurrence (n = 9) after DMAB treatment. Scale bar, 50 μm. Statistical table of 23 patients with GCTB by POSTN staining intensity. Significance was determined by a fisher exact test. *P < 0.05; **P < 0.01; ***P < 0.001 and ****P < 0.0001. All the results were obtained from three independent experiments. Values are presented as mean ± SD.

We further investigated the gene expression patterns of GCTB cells in the DMAB-treated and untreated samples (**Figure 5A**). The analysis revealed that activator protein 1 transcription factors (c-Fos, c-Jun, and FOSB) were significantly upregulated in DMAB-treated GCTB cells (**Figure 5B**). Furthermore, chromatin immunoprecipitation (ChIP) assay results demonstrated that c-FOS binds to the POSTN promoter in GCTB (**Figure 5C**).

POSTN is a secreted extracellular matrix protein, which is usually associated with poor prognosis in cancers. Indeed, we found that high POSTN expression was associated with poor prognosis in kidney papillary cell carcinoma, liver cancer, and lung cancer (**Supplementary Figures S5A–C**). Furthermore, POSTN is highly expressed in bone metastasis of prostate cancer and breast cancer (Figure S5D, S5E, and S5F). To determine the correlation between POSTN expression and relapse of GCTB in patients receiving DMAB, we stained DMAB-treated GCTB clinical specimens with POSTN with or without recurrence. POSTN expression levels were upregulated in DMAB-treated patients with relapse. Tumor relapse was positively correlated with POSTN staining (**Figure 5D**). These results demonstrate that POSTN expression levels have important prognostic significance for GCTB patients treated with DMAB.

## Discussion

**Figure 6:**
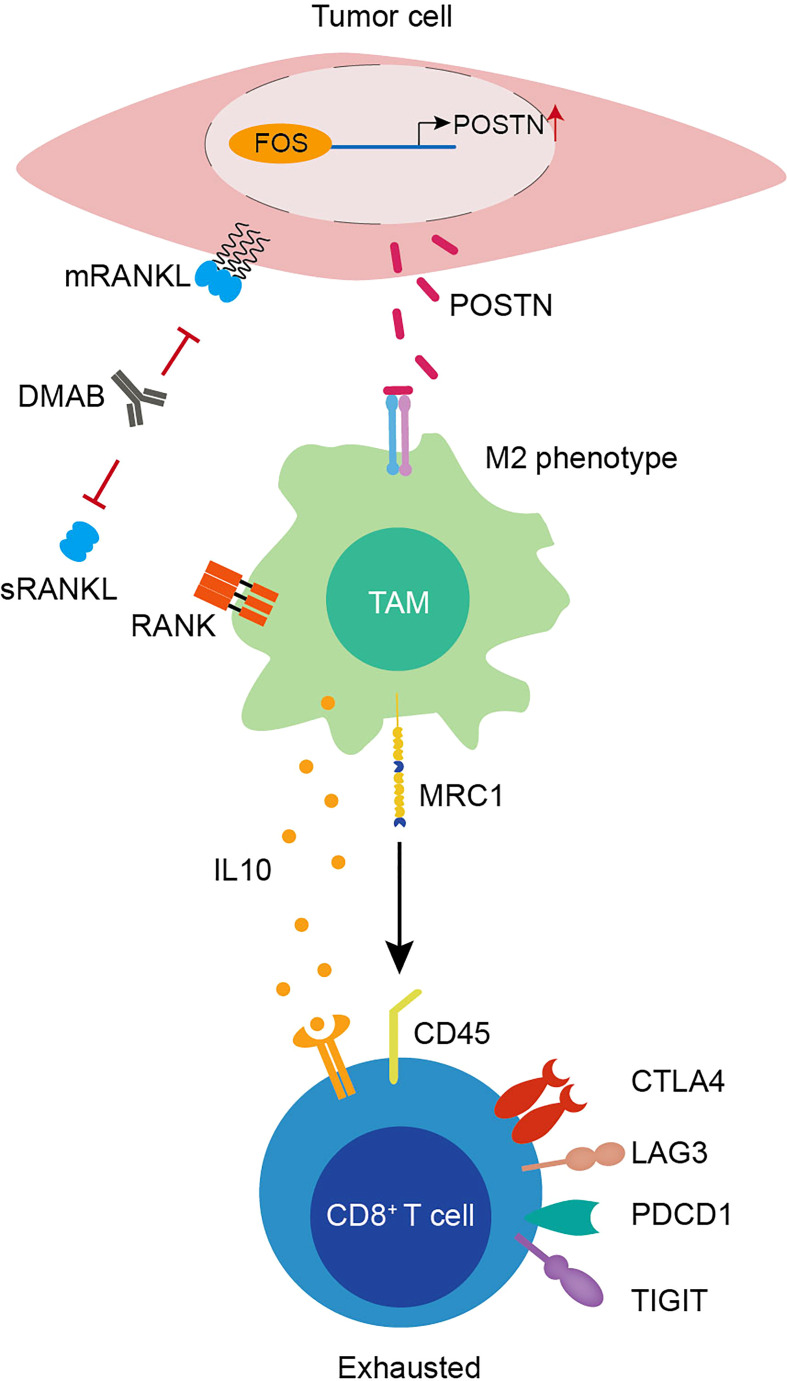
Graphical illustration of T-cell exhaustion in DMAB-treated GCTB based on bioinformatic prediction.

Little is known about the mechanisms mediating the adverse effects of DMAB therapy in patients with GCTB. Here, we depicted the cellular landscape and transcriptional profiles of GCTB with or without DMAB therapy, encompassing immune cells and tumor cells. We revealed an unappreciated T-cell dysfunction in DMAB-treated GCTB, whereby T cells undergo a transition from activated to exhausted T cells. Moreover, we identified the upregulation of POSTN as a possible mechanism of T cell exhaustion and proposed its relevance to recurrence in patients with GCTB treated with DMAB (**Figure 6**). This study provides a comprehensive cellular interaction atlas of DMAB-treated GCTB and a framework for improving DMAB therapy in the future.

DMAB may have dual functions in immunomodulation. Previous preclinical data showed that DMAB treatment increased tumor-infiltrating lymphocytes (TILs) in breast cancer, indicating that DMAB might improve the response to immunotherapy in patients with breast cancer. Based on this observation, new clinical trials for DMAB combined with immune checkpoint inhibitors should be conducted for breast cancer (34). In the present study, we used scRNA-seq combined with immunostaining to demonstrate that DMAB induces T-cell exhaustion in GCTB. In chronic infections and cancer, T cell exhaustion is caused by long-term exposure to persistent antigen (35).

To investigate the causes of T-cell exhaustion in GCTB with DMAB therapy, we analyzed the transcriptome of immune cells from GCTB and found that CD8^+^ T cell exhaustion integrates stimuli from altered tumor microenvironments. CD8^+^ T cells treated with DMAB showed increased costimulatory markers, indicating that DMAB may promote the continuous exposure of CD8^+^ T cells to the persisting antigen, especially in patients who receive DMAB at a higher frequency and longer time. Additional signals from inhibitory receptors (PD1, LAG3, and CTLA4) may also contribute to CD8^+^ T cell exhaustion in GCTB. Our understanding of the mechanisms by which inhibitory receptors control T-cell exhaustion in GCTB remains unclear. Our scRNA-seq data revealed that these inhibitory receptors might cause transient intracellular attenuation of positive signals and the induction of inhibitory gene expression to facilitate T-cell exhaustion. Because T cell exhaustion is reversible, agents that target inhibitory receptors may improve DMAB therapy.

The efficacy of DMAB in GCTB may be correlated with the M1-M2 TAM polarization status. RANK-RANKL induces macrophage differentiation into osteoclasts. RANKL also triggers M1 polarization of macrophages during bone formation (36). However, the role of RANK-RANKL in TAM remains unclear. A previous study showed that the loss of RANK signaling in mouse breast cancer cells reduces TAM and tumor-associated neutrophil infiltration (34). In our study, we showed that M1 and M2 type TAMs coexist in GCTB, whereas the majority of TAMs exhibit an M2-like phenotype. This is consistent with previous reports (37–40). Intriguingly, inhibition of RANK-RANKL signaling by DMAB accompanied by M1 TAM peaking early and switching towards M2 TAM in the TME of GCTB may facilitate the escape of T-cell immune surveillance by GCTB cells. Furthermore, M2-like TAMs were composed of four distinct subsets with different transcriptome profiles. However, the four subsets have overall similarity to M2 TAMs, such as secretion of IL-10, which is an important extrinsic cytokine involved in T cell exhaustion. Although the M1 to M2 phenotypic transition of TAM can be promoted by GCTB-secreted POSTN, the precise origin and function of M2-like TAM in GCTB still require further investigation.

It has been well demonstrated that POSTN expression is deregulated in malignant transformation. High POSTN expression levels are usually associated with aggressive tumor behavior and poor prognosis in cancer (41–44). For example, using immunohistochemical analyses, Hu et al. showed that POSTN expression was higher in osteosarcoma than in osteochondroma. Osteosarcoma patients with high levels of POSTN had a worse prognosis than those with low POSTN expression (45). Our findings revealed that POSTN expression was correlated with the recurrence of GCTB in patients receiving DMAB therapy, suggesting that POSTN levels could be a useful prognostic biomarker in GCTB. Recent studies have also shown that POSTN plays an important role in cancer treatment resistance. Liu et al. showed that POSTN confers gemcitabine resistance in pancreatic cancer cells (46). Hu et al. demonstrated the effect of POSTN on cisplatin resistance in NSCLC cells (47). Sung et al. also found a correlation between cisplatin resistance and POSTN in patients with ovarian cancer (48). Recombinant POSTN promotes resistance to carboplatin and paclitaxel in ovarian cancer cells (49). Taken together, these findings, including our work, suggest that POSTN targeting could be a new therapeutic approach to overcome DMAB therapy failure in GCTB.

## Data availability statement

The data presented in the study are deposited in the NCBIGene Expression Omnibus database (https://www.ncbi.nlm.nih.gov/), accession number GSE212341.

## Ethics statement

The studies involving human participants were reviewed and approved by IEC for Clinical Research and Animal Trials of the First Affiliated Hospital of Sun Yat-sen University. The patients/participants provided their written informed consent to participate in this study.

## Author contributions

MY performed the scRNA-seq analyses, IHC staining assays, and wrote the manuscript. FW performed the IHC staining assays. GL performed ChIP-qPCR assays. MC interpreted the data and revised the manuscript. WZ and CZ designed the experiments, interpreted the data, wrote the manuscript, and provided supervision. All authors contributed to the article and approved the submitted version.

## Funding

This work was supported by the National Natural Science Foundation of China (81972507, 81972651, and 82172698), and CSCO-GCT research Project (Y-2020GCT-003).

## Acknowledgments

We thank Dr. Meng Zhao for valuable suggestions.

## Conflict of interest

The authors declare that the research was conducted in the absence of any commercial or financial relationships that could be construed as a potential conflict of interest.

## Publisher’s note

All claims expressed in this article are solely those of the authors and do not necessarily represent those of their affiliated organizations, or those of the publisher, the editors and the reviewers. Any product that may be evaluated in this article, or claim that may be made by its manufacturer, is not guaranteed or endorsed by the publisher.
