# Supplementary material for: Single-cell transcriptome analysis reveals T-cell exhaustion in denosumab-treated giant cell tumor of bone
Source: Front Immunol. 2022 Sep 12;13:934078. doi: 10.3389/fimmu.2022.934078 (PMC9510370; doi:10.3389/fimmu.2022.934078)
Supplement: Supplementary file 1 [file DataSheet_1.docx]

Supplementary Material

# Supplementary Figures and Tables

## Supplementary Figures

**
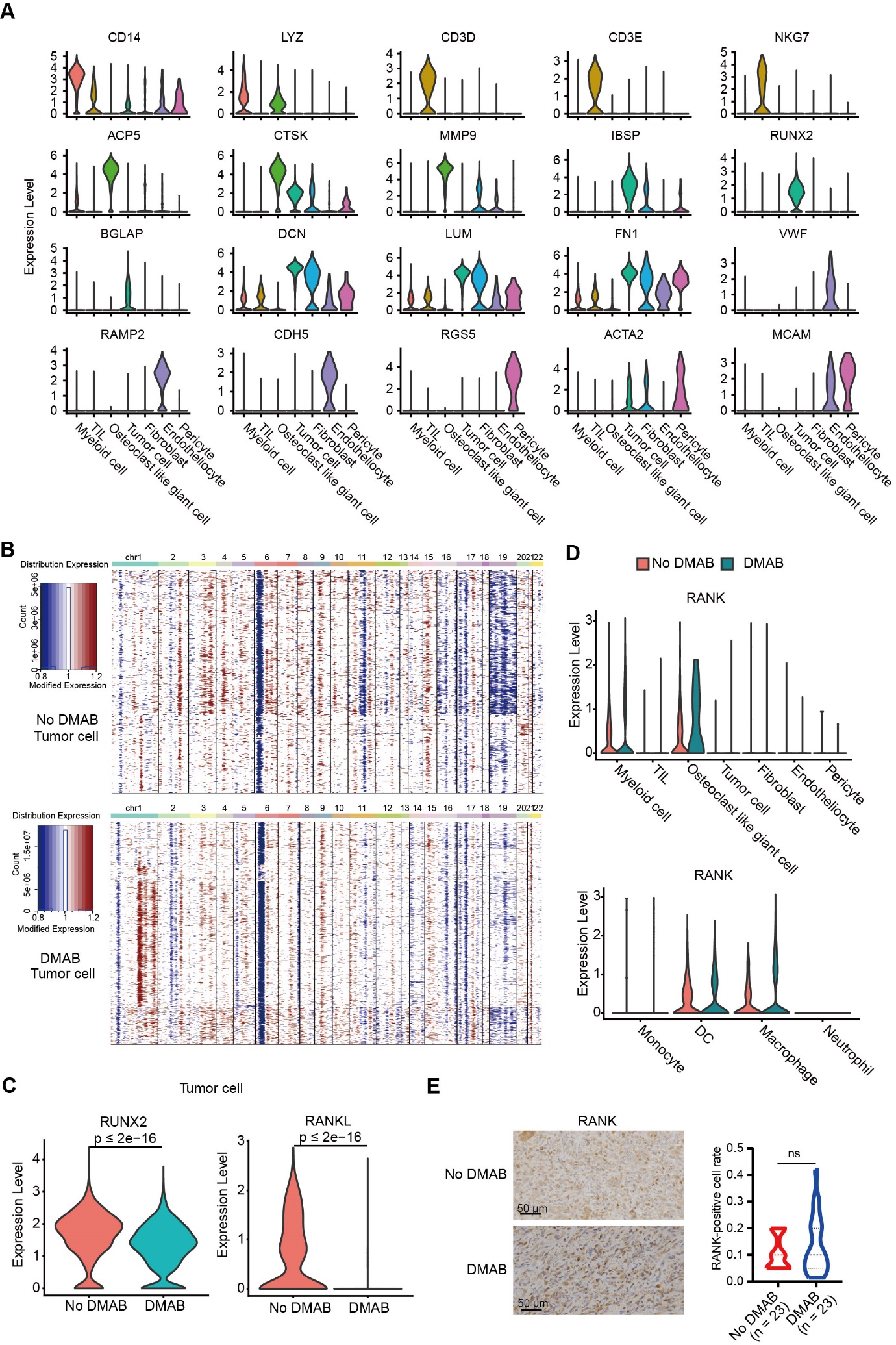
**

Figure S1. Single-cell RNA sequencing (ScRNA-seq) profiling of the ecosystem in untreated and Denosumab (DMAB)-treated giant cell tumor of bone (GCTB) samples, related to Figure 1. **(A)** Violin plot showing the expression levels of different marker genes across the indicated cell types. **(B)** Heatmap showing large-scale copy number variations (CNVs) for GCTB tumor cells, inferred from the single-cell RNA-seq data. Red, amplifications; blue, deletions. **(C)** Violin plot showing the expression of RUNX2 and RANKL in tumor cells in No DMAB (red) and DMAB (blue) samples. The p values are calculated by Student’s t test. **(D)** Violin plot showing the expression of RANK in GCTB (upper) and in myeloid cell subtypes (lower). Red, No DMAB; blue, DMAB. **(E)** Representative images of immunohistochemistry (IHC) staining in formalin-fixed paraffin-embedded (FFPE) tissues, indicating RANK^+^ cells in unpaired patients with GCTB with (n = 23) or without (n = 23) DMAB. Scale bar, 50 μm. Violin plot presenting the fractions of RANK^+^ cells in unpaired No DMAB and DMAB samples based on IHC staining results. Statistical analyses are Mann-Whitney tests.


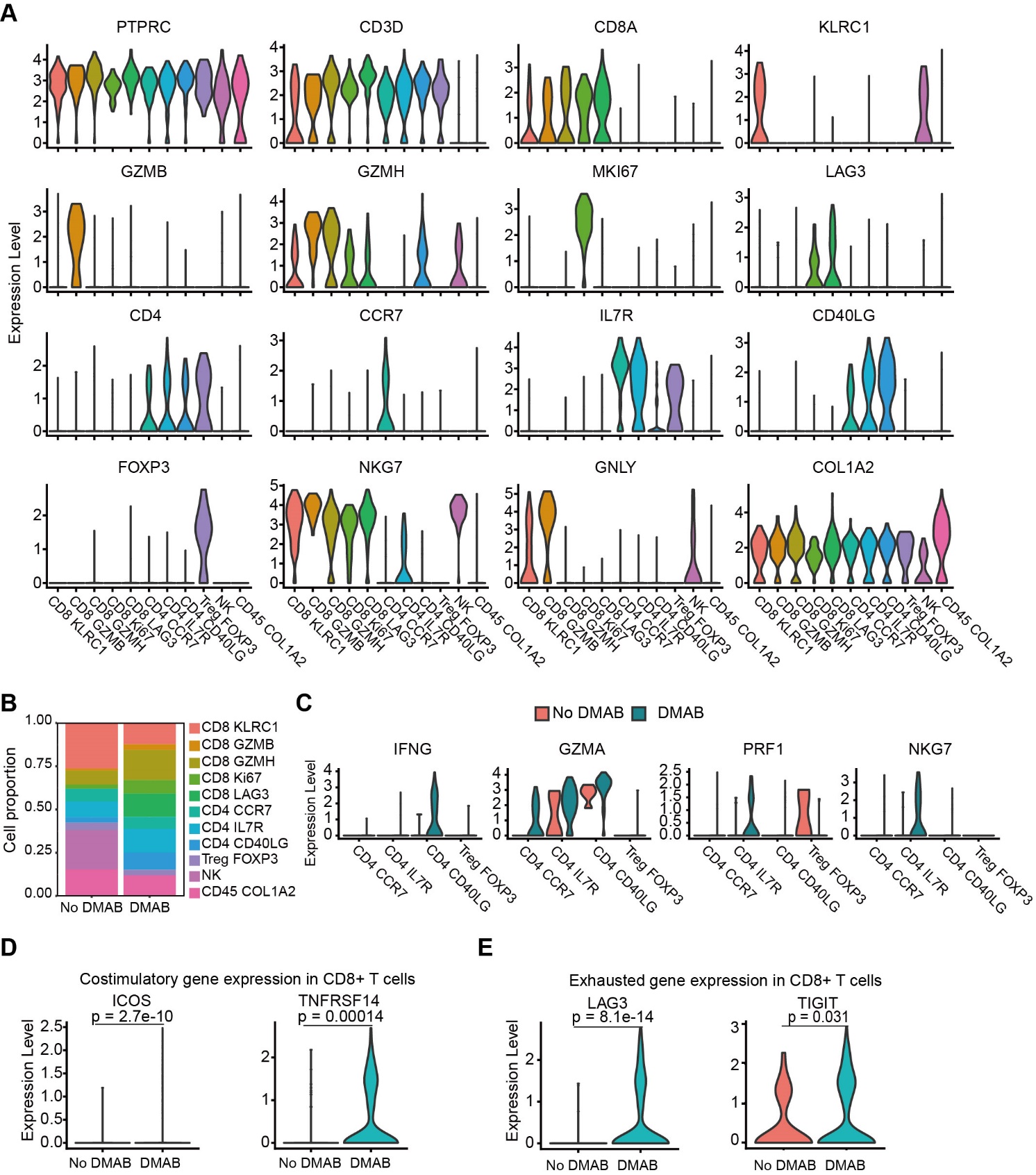


Figure S2. DMAB treatment promotes exhaustion of CD8^+^ T cells, related to Figure 2. **(A)** Violin plot showing the expression levels of different marker genes in each subtype of tumor infiltrating lymphocytes (TILs). **(B)** Histogram showing the proportion of tumor-infiltrating lymphocytes (TILs) subgroups in No DMAB and DMAB-treated samples. **(C)** Violin plot showing the expression levels of selected cytotoxic genes in CD4^+^ T cell subtypes. Red, No DMAB; blue, DMAB. **(D)** Violin plot showing the expression of selected costimulatory genes in CD8^+^ T cells from the No DMAB (red) and DMAB (blue) samples. The p values are calculated by Student’s t test. **(E)** Violin plot showing the expression of selected exhausted genes in CD8^+^ T cells from the No DMAB (red) and DMAB (blue) samples. The p values are calculated by Student’s t test.


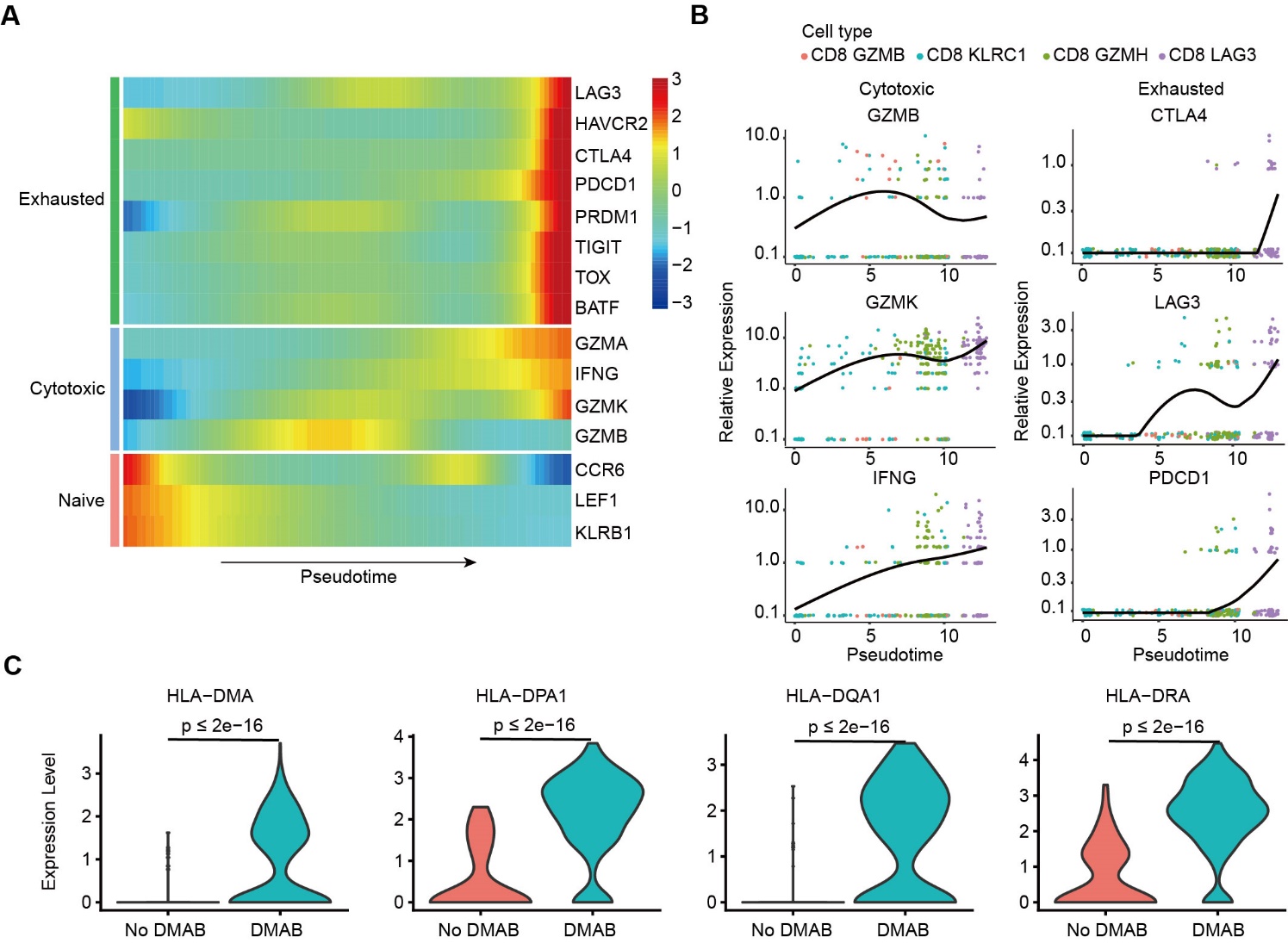


Figure S3. The dynamic cell transitions and immune states of CD8^+^ T cells in GCTB, related to Figure 3. **(A)** Heatmap showing the dynamic changes in gene expression along the pseudotime. The selected genes are associated with naive, cytotoxic or exhausted states of CD8^+^ T cells. **(B)** Plots showing the trends of expression profiles of selected genes associated with cytotoxic or exhausted in different CD8^+^ T cell subtypes along the pseudotime. **(C)** Violin plot showing the expression of selected MHC-Ⅱ in CD8^+^ T cells from the No DMAB (red) and DMAB (blue) samples. The p values are calculated by Student’s t test.


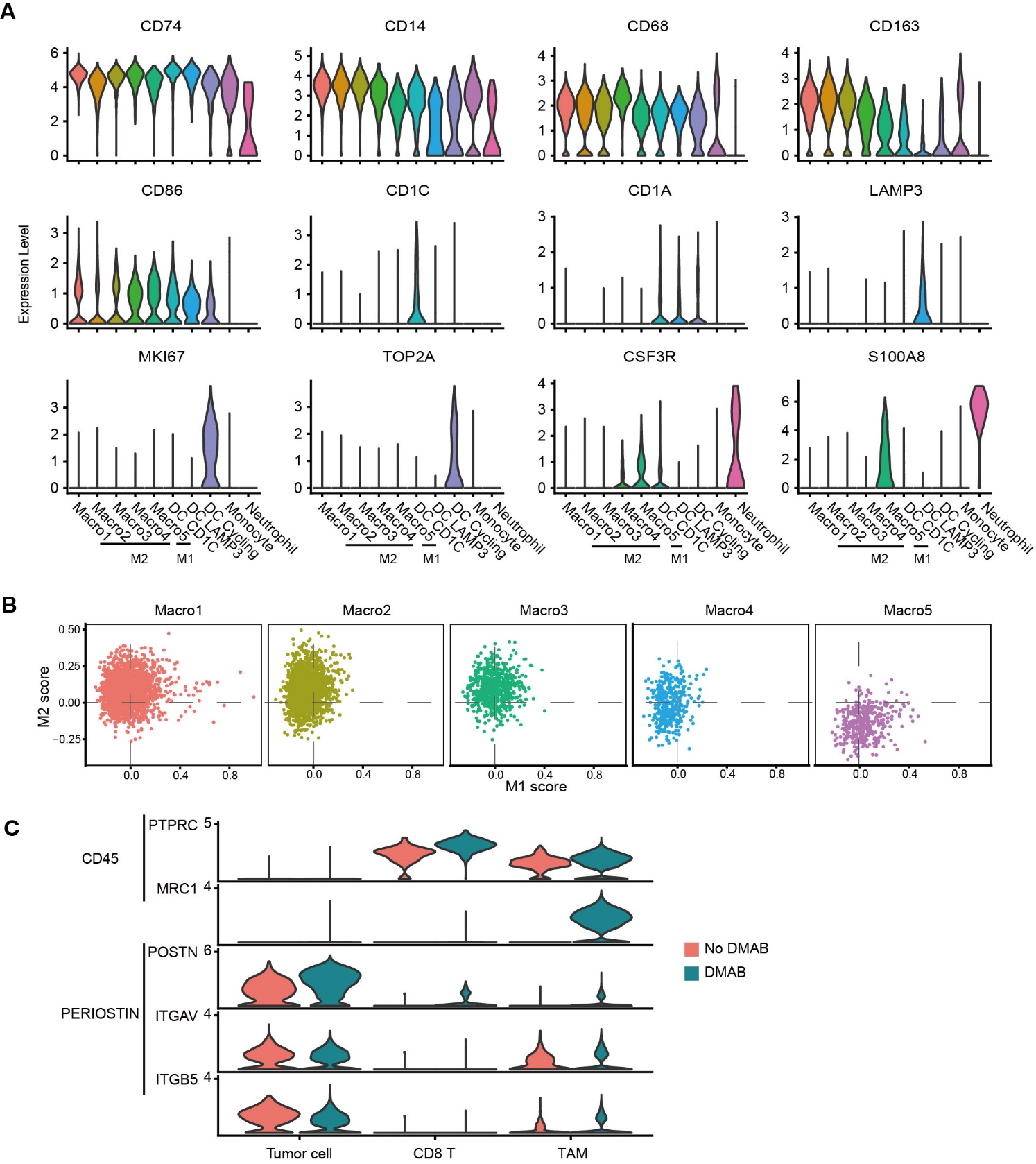


Figure S4. DMAB treatment promotes the M2 subtype of tumor associated macrophages (TAMs) via POSTN, related to Figure 4. **(A)** Violin plot showing the expression levels of different marker genes in each subtype of myeloid-derived cells. **(B)** Scatterplot showing the M1 and M2 scores of TAM subtypes. **(C)** Violin plot showing the expression distribution of signaling genes involved in the inferred signaling network, including Periostin (POSTN) and CD45 signaling. Red, No DMAB; blue, DMAB.

**
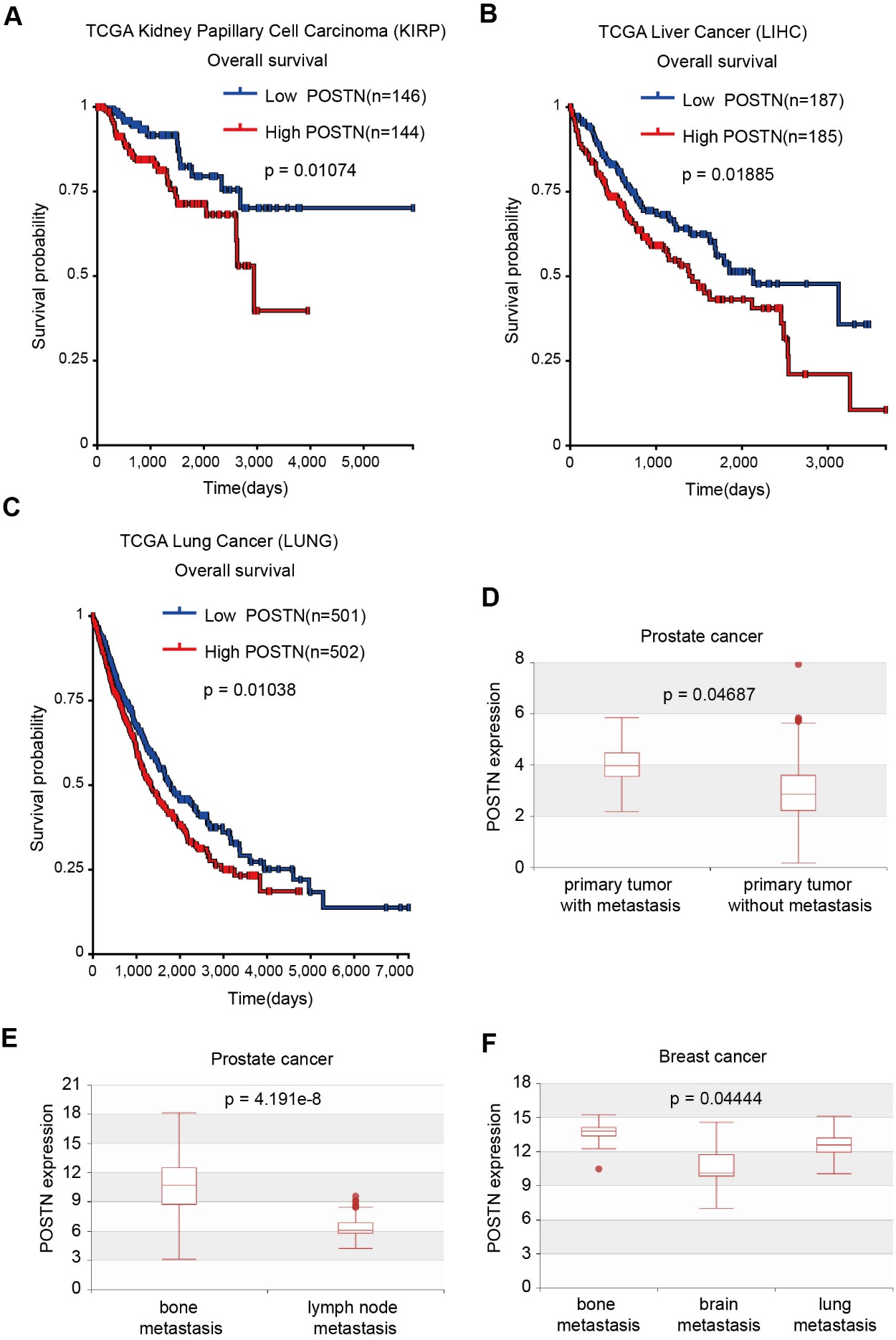
**

Figure S5. The expression of POSTN in cancers, related to Figure 5. **(A)** Kaplan-Meier overall survival curve of kidney papillary cell carcinoma (KIRP) by POSTN expression based on 21 RNA-seq datasets from The Cancer Genome Atlas (TCGA). **(B)** Kaplan-Meier overall survival curve of liver cancer (LIHC) by POSTN expression based on 19 RNA-seq datasets from TCGA. **(C)** Kaplan–Meier overall survival curve of lung cancer (LUNG) by POSTN expression based on 15 RNA-seq datasets from TCGA. All analyses were performed on USCS Xena (http://xena.ucsc.edu/). The patients were divided into high expression group and low expression group according to the median expression. **(D)** Box plot showing the expression of POSTN in primary tumor of prostate cancer with or without metastasis based on TCGA dataset (Dataset ID: TCGA-PRAD). **(E)** Box plot showing the expression of POSTN in metastatic tumor of prostate cancer with bone metastasis or lymph node metastasis based on dataset GSE74685. **(F)** Box plot showing the expression of POSTN in metastatic tumor of breast cancer with bone metastasis, brain metastasis or lung metastasis based on dataset GSE14017. All analyses were performed on HCMDB (Human Cancer Metastasis Database).

## Supplementary Table

Table S1. Signature related gene sets used in calculating cell scores analysis. Related to Figures 2, 4 and S4.

| Co-stimulatory | Exhausted | M1 Polarization | M2 Polarization | Anti-inflammatory |
| --- | --- | --- | --- | --- |
| ICOS | CTLA4 | MARCO | ARG2 | IL1RN |
| CD28 | HAVCR2 | FCGR1A | IL10 | IL10 |
| CD226 | LAG3 | CD80 | FCGR2A | TGFB1 |
| TNFRSF14 | PDCD1 | IL23A | FCGR2B | TNFRSF1A |
| TNFRSF25 | TIGIT | CXCL9 | CD163 | TNFRSF1B |
| TNFRSF9 |  | CXCL10 | FCER2 | IL1R2 |
|  |  | CXCL11 | CD200R1 | IL18BP |
|  |  | CD86 | PDCD1LG2 |  |
|  |  | IL1A | CD274 |  |
|  |  | IL1B | MARCO |  |
|  |  | IL6 | CSF1R |  |
|  |  | TNF | MRC1 |  |
|  |  | CCL5 | IL1RN |  |
|  |  | IRF5 | IL1R2 |  |
|  |  | IRF1 | IL4R |  |
|  |  | CD40 | CCL4 |  |
|  |  | IDO1 | CCL13 |  |
|  |  | KYNU | CCL20 |  |
|  |  | CCR7 | CCL17 |  |
|  |  |  | CCL18 |  |
|  |  |  | CCL22 |  |
|  |  |  | CCL24 |  |
|  |  |  | LYVE1 |  |
|  |  |  | VEGFA |  |
|  |  |  | VEGFB |  |
|  |  |  | VEGFC |  |
|  |  |  | CTSA |  |
|  |  |  | CTSB |  |
|  |  |  | CTSD |  |
|  |  |  | TGFB1 |  |
|  |  |  | TGFB2 |  |
|  |  |  | TGFB3 |  |
|  |  |  | MMP14 |  |
|  |  |  | MMP19 |  |
|  |  |  | MMP9 |  |
|  |  |  | CLEC7A |  |
|  |  |  | WNT7B |  |
|  |  |  | TNFSF12 |  |
|  |  |  | TNFSF8 |  |
|  |  |  | CD276 |  |
|  |  |  | MSR1 |  |
|  |  |  | FN1 |  |
|  |  |  | IRF4 |  |
